# Sezary syndrome initially presenting as pityriasis rubra pilaris: Clinicopathologic study of 3 cases

**DOI:** 10.1016/j.jdcr.2023.12.003

**Published:** 2023-12-27

**Authors:** Mouaz Alsawas, Eyas Alzayadneh, Eric Mou, Vincent Liu

**Affiliations:** a Department of Pathology, University of Iowa, Iowa City, Iowa; b Division of Hematology and Oncology, Department of Internal Medicine, University of Iowa, Iowa City, Iowa; c Departments of Dermatology and Pathology, University of Iowa, Iowa City, Iowa

**Keywords:** biologic immunomodulatory agent, cutaneous T-cell lymphoma, dermatopathology, diagnosis, disease evolution, pityriasis rubra pilaris, Sezary syndrome

## Introduction

Pityriasis rubra pilaris (PRP) and Sezary syndrome (SS) are 2 rare diseases traditionally thought to be distinct in nature, but which clinically can share an erythrodermic cutaneous presentation. PRP is a rare inflammatory papulosquamous disorder that classically presents with salmon-colored patches and plaques with characteristic islands of sparing, follicular accentuation, and palmoplantar keratoderma, uncommonly evolving to erythroderma.^1^ In contrast, SS is a type of cutaneous T-cell lymphoma (CTCL) that manifests as erythroderma with associated leukemic Sezary cells in blood and often lymph nodes.^2^

To date, no clear relationship between PRP and SS has been well documented, although occasional reports of paraneoplastic PRP as well as rare reports of SS presenting as PRP exist.^3^ In this study, we interrogated our institutional CTCL registry for patients with SS with previous history of PRP, and herein, we present the clinicopathologic profiles of the 3 patients, exploring a possible relationship between these 2 rare diseases.

## Methods

Following institutional review board approval, we queried the cutaneous lymphoma registry at a tertiary care center, searching for all cases of CTCL with a history of PRP since 2000. We identified all patients who initially presented with a clinicopathologic picture of PRP, which evolved to SS,^4^ as confirmed by clinical and pathologic parameters, including histopathologic, immunophenotypic, and molecular T-cell clonality studies of skin biopsy specimens, lymph node biopsy specimens, and peripheral blood. Medical records were reviewed in detail, collecting data on patient age, sex, clinical presentation, treatment regimens, therapy response, course, and outcomes. Glass slides of skin biopsies were reviewed, tabulating histopathologic and immunohistochemical findings.

## Cases

**Fig 1:**
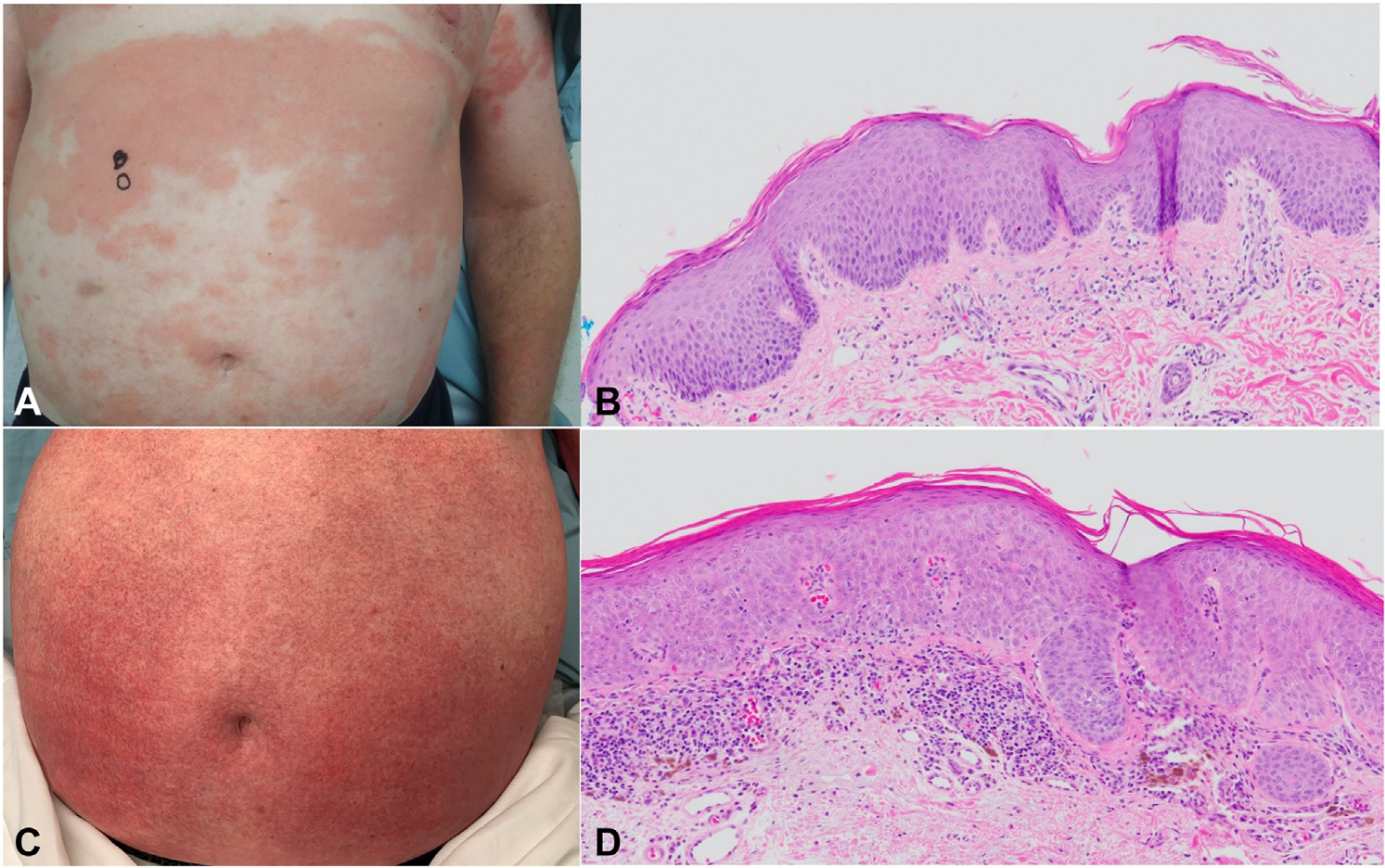
Clinical and pathologic findings for patient #1. **A,** PRP phase presenting confluent orange-red patches with islands of sparing covering the abdomen. **B,** Skin biopsy (H&E; 100×) demonstrating patterned parakeratosis in horizontal and vertical dimensions, overlying mildly spongiotic epidermis dotted by occasional lymphocytic exocytosis, with subjacent mild superficial perivascular lymphocytic inflammation consistent with pityriasis rubra pilaris. **C,** SS phase (5 years after PRP phase) showing erythroderma with fine scaling over abdomen. **D,** Skin biopsy (H&E; 100×) shows lichenoid atypical lymphocytic infiltrate with epidermotropism into compactly parakeratotic epidermis, consistent with SS.

Three patients, all men (100 %) were identified, exhibiting an age range of 51 to 72 years at initial PRP diagnosis (Table I). All patients initially presented with pruritic erythematous patches and/or thin plaques, collectively starting on the lower extremities or trunk, with islands of sparing, subsequently spreading to other areas of the body, eventuating in erythroderma (Fig 1, *A*). All patients were treated with topical corticosteroids as well as with at least 1 systemic agent, with 2 of the 3 patients receiving acitretin. All 3 patients experienced therapy response deemed to be only partial, variable, and transient. Two patients (patients 1 and 2) received additional biologic therapy, without significant improvement (Table I).

**Table I:** Clinicopathologic characteristics at pityriasis rubra pilaris presentation for included series of patients

Patient	Sex	Age of onset (y)	Clinical features	Dermatopathologic findings	Treatment		Response
					Topical	Systemic	
1	Male	52	Scaly, dry skin of trunk, progressively more erythematous and pruritic, maturing into diffuse pruritic erythema with islands of sparing of the trunk, spreading to the legs with swelling, and later to the head	Parakeratosis in checkerboard pattern, mild spongiosis, mild irregular acanthosis, rare lymphocytic exocytosis with rare dyskeratosis, and mild superficial perivascular lymphocytic inflammation	Corticosteroids	Acitretin Methotrexate Adalimumab Cyclosporine Ustekinumab Apremilast	Partial, variable, transient
2	Male	51	Chronic pruritic papulosquamous eruption with islands of sparing, initially on lower extremities spreading to most of the body, including face	Hyperkeratosis and parakeratosis with hypogranulosis and epidermal spongiosis with superficial dermal and perivascular lymphoplasmacytic inflammation	Corticosteroids Calcipotriene	Prednisone Mycophenolate Methotrexate Adalimumab Secukinumab Cyclosporine	Partial, variable, transient
3	Male	72	Erythema of chest and back spreading from the torso to the bilateral upper extremities, with islands of sparing	Orthokeratosis with focal parakeratosis, occasional follicular plugging, acanthosis and psoriasiform hyperplasia with focal spongiosis, interface dermatitis, and superficial perivascular lymphocytic infiltrate	Corticosteroids	Prednisone Methotrexate Acitretin	Partial, variable, transient

Dermatopathologic analysis of skin biopsies available for 3 of the patients corresponding to their PRP clinical periods generally showed variably checkerboard patterns of parakeratosis, variably prominent spongiosis, and mild-to-moderate superficial perivascular lymphocytic infiltrates, with variable psoriasiform hyperplasia in 2 cases (patients 1 and 2). Lymphocytic migration into the epidermis was noted in one case (patient 1) 2 years before the diagnosis of SS (Fig 1, *B*).

SS was diagnosed between 5 and 6 years (mean, 5.3 years) after initial diagnosis of PRP in the 3 patients (Table II). All patients clinically presented with erythroderma at primary diagnosis of SS (Fig 1, *C*). Skin biopsies for all patients exhibited features compatible with SS, including lichenoid and superficial perivascular, predominantly atypical T-lymphocytic infiltrates with variable epidermotropism (Fig 1, *D*).

**Table II:** Clinicopathologic characteristics at Sezary syndrome presentation for included series of patients

No.	Age of diagnosis (no. of years after PRP diagnosis)	Clinical features	Pathologic features			Radiographic staging	Systemic treatment	Response
			Skin biopsy	Peripheral blood	Lymph node			
1	57 (5)	Erythroderma; flaring after 2 injections of adalimumab, and methotrexate (×5 mo)	Monoclonal CD4^+^ lymphocytes in superficial perivascular, lichenoid, and focally intraepidermal arrangement, with admixed eosinophils, plasma cells, and histiocytic giant cells, set in background parakeratotic, mildly spongiotic, irregular acanthosis	Involved by Sezary syndrome: immunophenotypically aberrant CD3^+^/CD4^+^/CD7^−^ T cells (>1000/mm^3^) with same clone as in skin and LN biopsies	Right inguinal LN: involved by Sezary syndrome with same clone as in skin biopsy and in PB	Several prominent axillary LNs bilaterally, many with fatty hilum; largest right axillary LN, with background nonspecific enlarged LNs in the axilla, mediastinum, and inguinal regions	Photopheresis Bexarotene Mogamulizumab Allogenic stem cell transplant	Ongoing remission (2 y posttransplant; age 61 y)
2	57 (6)	Erythroderma, progressive papular eruption, pruritus	Monoclonal epidermotropic, lichenoid and superficial perivascular atypical T-lymphocytic infiltrate	T cells with increased CD4/CD8 ratio (12.7), with same clone as in skin and LN biopsies	Right inguinal LN positive for T-cell lymphoma with same clone as in skin biopsy and in PB	Bilateral axillary and inguinal lymphadenopathy	Bexarotene Photopheresis Romidepsin Pralatrexate	Partial response; stable disease (age 61 y)
3	77 (5)	Progressive erythroderma, pruritus, and scaling gradually worsened over 8-12 mo, with development of left inguinal lymphadenopathy, persisting despite penicillin for Group B strep infection	Monoclonal epidermotropic, lichenoid atypical T-lymphocytic infiltrate	Involved by Sezary syndrome: immunophenotypically aberrant CD3^+^/CD4^+^/CD7^−^/CD26^−^ T cells (>1000/mm^3^) with same clone as in skin and LN biopsies	Right inguinal LN positive for T-cell lymphoma with same clone as in skin biopsy and in PB	Mildly enlarged FDG avid left axillary and bilateral inguinal LNs, concerning for lymphoma	Mogamulizumab	Complete response (age 79 y)

*FDG*, Fluorodeoxyglucose; *LN*, lymph node; *PB*, peripheral blood; *PRB*, pityriasis rubra pilaris.

T-cell receptor gene rearrangement studies were performed on skin biopsies for each of the 3 patients at the time of first diagnostic biopsy of SS. These showed peaks in the Vg9 + Jg1.3/2.3, Vg1-8 + Jg1.3/2.3, and Vg1-8 regions, respectively. Moreover, for each of the 3 patients, the matching T-cell clone was identified for skin, lymph node, and blood. Peripheral blood flow cytometry and lymph node biopsies were all consistent with SS (Table II).

The 3 patients are alive and currently receiving different treatment regimens. One patient underwent hematopoietic stem cell transplant, another patient is receiving romidepsin and extracorporeal photopheresis, and the third patient is receiving treatment with mogamulizumab.

## Discussion

This study presents 3 patients who were initially diagnosed with PRP, refractory to multiple topical and systemic treatment regimens, who were later diagnosed with SS. This apparent evolution from 1 rare inflammatory dermatosis into a distinct malignant neoplastic disorder merits careful consideration of a potential relationship between PRP and SS.

All possible explanations for seeming transition from PRP to SS must be entertained, including the possibility that the initial diagnosis of PRP was unfounded. With an incidence rate of 1 in 5000,^5^ PRP is a rare papulosquamous inflammatory dermatosis. PRP typically presents as a follicular hyperkeratotic papular eruption of red-orange color involving the upper part of the body, spreading to erythroderma in some cases. Classical adult-onset, atypical adult-onset, classical juvenile-onset, circumscribed juvenile, and atypical juvenile-onset types are recognized. Microscopically, PRP classically displays alternating checkerboard-like orthokeratosis and parakeratosis and may demonstrate follicular plugging with a concomitant superficial perivascular lymphocytic infiltrate.^1^ All 3 of the included patients met these clinical and pathologic criteria during the initial PRP phase of disease (Table I).

Similarly, the validity of the SS diagnosis deserves scrutiny. According to the World Health Organization-European Organization for Research and Treatment of Cancer cutaneous lymphoma classification scheme, SS is defined as the triad of pruritic erythroderma, generalized lymphadenopathy, and clonally related neoplastic T cells with cerebriform nuclei (Sezary cells) in the skin, lymph nodes, and peripheral blood.^4^ To qualify for blood involvement, detection of clonally related neoplastic T cells in skin and peripheral blood, plus either an absolute Sezary cell count of >1000/μL or an expanded CD4^+^ T-cell population resulting in a CD4/CD8 ratio ≥ 10, CD4^+^/CD7^−^ cells ≥ 30%, or CD4^+^/CD26^−^ cells ≥ 40%, are required.^4^ All 3 of the included patients during the SS diagnosis phase satisfy these clinical and pathologic thresholds (Table II).

Having fulfilled diagnostic criteria for both an initial PRP phase and a subsequent SS phase, possible explanations for a relationship between PRP and SS may be explored. One interpretation for this observation would regard PRP as a paraneoplastic phenomenon for the underlying SS. To date, the literature has documented at least 19 cases in which PRP has been identified as a paraneoplastic syndrome associated with lung, breast, prostate carcinomas, as well as hematologic malignancies.^6^, ^7^, ^8^ In the majority of these cases, initial treatment resistance was observed, followed by improvement or resolution after treatment of the concurrent malignancy.^8^

The lack of concurrent, distinctly PRP and SS features in our patients, however, favors 1 rather than 2 processes occurring at any given time. As such, 2 scenarios may be considered: (1) 2 successive, separate phases of initial PRP followed by SS or (2) SS initially mimicking PRP.^3^ As comprehensive immunophenotypic and molecular analysis of skin and blood was not exhaustively performed during the PRP phase, distinction between these 2 scenarios may not be feasible. The value of peripheral blood studies in patients with erythroderma in distinguishing SS from inflammatory disorders has been emphasized in the literature.^9^ Flow cytometry of peripheral blood abnormalities, including an elevated CD4/CD8 ratio and aberrant expression of CD26, CD27, and CD7, can be particularly helpful in suggesting SS, along with clues such as peripheral eosinophilia, and T-cell clonality.^9^

The intersection between CTCL and dermatitis classically focuses on the protean clinical manifestations of CTCL, which can resemble inflammatory dermatoses^10^^,^^11^; however, recent literature has drawn attention to patients with psoriasis and atopic dermatitis (AD) treated with immunomodulatory medications who were subsequently diagnosed with mycosis fungoides.^12^, ^13^, ^14^, ^15^ To date, however, no similar relationship between PRP and mycosis fungoides (MF) or SS has been clearly documented. A recent systematic review of the literature^15^ profiled 23 patients with AD treated with dupilumab in whom MF or SS developed subsequently. However, given the clinical and pathologic similarity between AD and MF or SS, it remains uncertain whether these patients truly evolved from AD to MF or SS during dupilumab treatment or if the underlying skin condition was actually MF or SS from the beginning and became apparent during dupilumab therapy. It is important to note that in our cohort, 2 out of 3 patients did receive biologic agents in treatment of presumed PRP, but neither patient received dupilumab.

Our case series of 3 patients each with an initial clinicopathologic diagnosis of PRP who evolved into SS offers novel insight into a possible connection between 2 rare diseases. Whether the initial “PRP” phase for our patients represented either true PRP or PRP-like SS would likely require comprehensive molecular profiling (eg, high-throughput sequencing) through time; nonetheless, this case series offers a PRP-like prelude phase (whether or not truly SS) as a novel potential harbinger of eventual SS. Given the challenge in diagnosis for both PRP and many cases of SS, continuous vigilance and deliberate integration of a constellation of clinical, histopathologic, immunopathologic, and molecular features in patients with refractory erythroderma are recommended. Extrapolating from accumulating experience of triggering, exacerbation, or revelation of CTCL during biologic therapy for presumptive AD and psoriasis, particular caution should be exercised in utilization of biologic agents in refractory PRP until SS has been excluded.

## Conflicts of interest

None disclosed.
